# Case Report: Consecutive Adrenal Cushing’s Syndrome and Cushing’s Disease in a Patient With Somatic *CTNNB1*, *USP8*, and *NR3C1* Mutations

**DOI:** 10.3389/fendo.2021.731579

**Published:** 2021-08-20

**Authors:** Mario Detomas, Barbara Altieri, Wiebke Schlötelburg, Silke Appenzeller, Sven Schlaffer, Roland Coras, Andreas Schirbel, Vanessa Wild, Matthias Kroiss, Silviu Sbiera, Martin Fassnacht, Timo Deutschbein

**Affiliations:** ^1^Department of Internal Medicine I, Division of Endocrinology and Diabetes, University Hospital Würzburg, University of Würzburg, Würzburg, Germany; ^2^Department of Diagnostic and Interventional Radiology, University Hospital Würzburg, University of Würzburg, Würzburg, Germany; ^3^Department of Nuclear Medicine, University Hospital Würzburg, University of Würzburg, Würzburg, Germany; ^4^Core Unit Bioinformatics, Comprehensive Cancer Center Mainfranken, University Hospital of Würzburg, University of Würzburg, Würzburg, Germany; ^5^Department of Neurosurgery, University Hospital Erlangen, Erlangen, Germany; ^6^Department of Neuropathology, University Hospital Erlangen, Erlangen, Germany; ^7^Institute of Pathology, University of Würzburg, Würzburg, Germany; ^8^Department of Internal Medicine IV, University Hospital Munich, Ludwig-Maximilians-Universität München, Munich, Germany; ^9^Medicover Oldenburg MVZ, Oldenburg, Germany

**Keywords:** Cushing’s syndrome, Cushing’s disease, hypercortisolism, glucocorticoid excess, USP8, CTNNB1, NR3C1

## Abstract

The occurrence of different subtypes of endogenous Cushing’s syndrome (CS) in single individuals is extremely rare. We here present the case of a female patient who was successfully cured from adrenal CS 4 years before being diagnosed with Cushing’s disease (CD). The patient was diagnosed at the age of 50 with ACTH-independent CS and a left-sided adrenal adenoma, in January 2015. After adrenalectomy and histopathological confirmation of a cortisol-producing adrenocortical adenoma, biochemical hypercortisolism and clinical symptoms significantly improved. However, starting from 2018, the patient again developed signs and symptoms of recurrent CS. Subsequent biochemical and radiological workup suggested the presence of ACTH-dependent CS along with a pituitary microadenoma. The patient underwent successful transsphenoidal adenomectomy, and both postoperative adrenal insufficiency and histopathological workup confirmed the diagnosis of CD. Exome sequencing excluded a causative germline mutation but showed somatic mutations of the β-catenin protein gene (*CTNNB1*) in the adrenal adenoma, and of both the ubiquitin specific peptidase 8 (*USP8*) and the glucocorticoid receptor (*NR3C1*) genes in the pituitary adenoma. In conclusion, our case illustrates that both ACTH-independent and ACTH-dependent CS may develop in a single individual even without evidence for a common genetic background.

## Introduction

Endogenous Cushing´s syndrome (CS) is a rare disorder with an incidence of 0.2–5.0 per million people per year (1, 2). The predominant subtype (accounting for about 80%) is adrenocorticotropic hormone (ACTH)-dependent CS. The vast majority of this subtype is due to an ACTH-secreting pituitary adenoma [so called Cushing´s disease (CD)], whereas ectopic ACTH-secretion (e.g. through pulmonary carcinoids) is much less common. In contrast, ACTH-independent CS can mainly be attributed to cortisol-producing adrenal adenomas. Adrenocortical carcinomas, uni-/bilateral adrenal hyperplasia, and primary pigmented nodular adrenocortical disease (PPNAD) may account for some of these cases as well (3, 4).

Coexistence of different subtypes of endogenous CS in single individuals is even rarer but has been described in few reports. These cases were usually observed in the context of prolonged ACTH stimulation on the adrenal glands, resulting in micronodular or macronodular hyperplasia (5–9). A sequence of CD and PPNAD was also described in presence of Carney complex, a genetic syndrome characterized by the loss of function of the gene encoding for the regulatory subunit type 1α of protein kinase A (*PRKAR1A*) (10). Moreover, another group reported the case of a patient with Cushing's disease followed by ectopic Cushing's syndrome more than 30 years later (8). To our knowledge, however, we here describe the first case report on a single patient with a cortisol-producing adrenocortical adenoma and subsequent CD.

## Case Description

**Figure 1:**
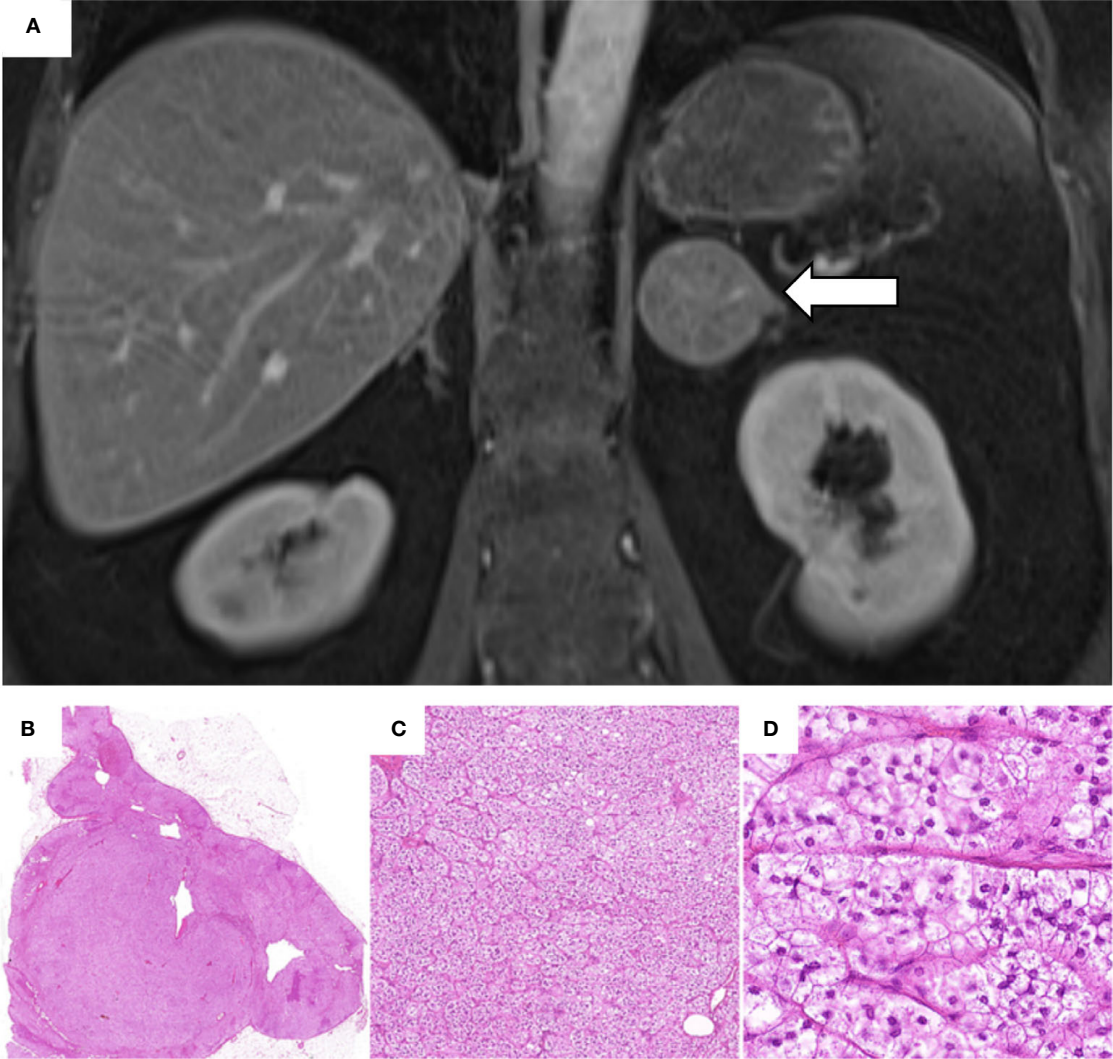
Abdominal magnetic resonance imaging performed in March 2014 and histological analysis of the adrenal adenoma. **(A)** Contrast-enhanced coronal T1-weighted MRI showing a 4.5 x 4.2 cm adenoma of the left adrenal gland (white arrow). **(B–D)** Histological investigation of the adrenal adenoma with hematoxylin and eosin staining: **(B)** Solitary, well circumscribed intra-adrenal mass confined to the gland; **(C)** Nested/fasciculated growth pattern of uniform tumor cells; **(D)** Tumor cells showing lipid-rich foamy cytoplasm with small uniform nuclei. Absent mitosis or necrosis. (H.E. 6.5x, 10.0x, 40.0x).

In March 2014, a 49-year old female underwent an abdominal magnetic resonance imaging (MRI) because of recurrent abdominal pain and irregular hypermenorrhea. This revealed the presence of a left-sided adrenal lesion of 4.5 x 4.2 cm (**Figure 1A**). Due to the persistence of menstrual cycle irregularities and the development of hirsutism, the general practitioner finally suspected a cortisol-producing adrenal tumor and the patient was, therefore, referred to our outpatient clinic.

At the time of her first examination at our outpatient clinic (January 2015), the patient reported muscle-weakness, asthenia, and hirsutism. She suffered from arterial hypertension and was treated with a triple anti-hypertensive therapy. Other comorbidities and medical therapies were not reported. Both parents had arterial hypertension, and the familial history was positive for breast cancer (mother). The physical examination revealed classical signs of CS such as centripetal obesity (body mass index: 45.2 kg/m^2^), striae rubrae, hirsuitism, facial plethora, skin atrophy, and multiple hematomas. Her glycated hemoglobin was slightly elevated (HbA1c 6.4%). The routine laboratory (including electrolytes) was unremarkable. The endocrine workup was conducted according to published guidelines (12), involved commercially available analytical procedures (i.e., the Immulite 2000 Xpi from Siemens for the analysis of ACTH and serum cortisol, a manual luminescence immunoassay from IBL for the analysis of salivary cortisol, and a manual radioimmunoassay from Immuntech for the analysis of urinary free cortisol) and indicated presence of ACTH-independent CS (as shown in **Table 1**). Of note, basal ACTH was repeatedly <10 ng/l (reference range 0-46 ng/l). Furthermore, there was no evidence for primary hyperaldosteronism or a catecholamine excess.

**Table 1:** Endocrine evaluation in our outpatient clinic (January 2015) and at our endocrine ward (April 2019).

Initial evaluation (January 2015) - diagnosis of ACTH-independent Cushing’s syndrome	Result	Reference range
ACTH (ng/l)	6.3	0 - 46
Basal serum cortisol (µg/dl)	**27.6**	5-25
Serum cortisol after 1 mg dexamethasone (µg/dl)	**16.3**	0 - 1.8
Late-night salivary cortisol (µg/dl)	**0.34**	0 - 0.15
24-hour urinary free cortisol (µg/24h)	**182**	8 - 70
Aldosterone (ng/l)	36	38 - 313
Plasma renin concentration (ng/l)	14	3 - 57
Plasma metanephrine (ng/l)	57.7	0 - 90
Plasma normetanephrine (ng/l)	59.3	0 - 200
Total testosterone (µg/l)	0.2	0 - 0.73
DHEA-S (µg/dl)	55	26 - 200
Androstendione (µg/l)	3.03	0.47 -2.68
17α-hydroxyprogesterone (µg/l)	1.5	0.3 - 3.6
**Follow-up evaluation (April 2019) – diagnosis of ACTH-dependent Cushing’s syndrome**	**Result**	**Reference range**
ACTH (ng/l)	**69.3**	0 - 46
Basal serum cortisol (µg/dl)	23.5	5-25
Serum cortisol after 1 mg dexamethasone (µg/dl)	**28.0**	0 - 1.8
Late-night salivary cortisol (µg/dl)	**0.21**	0 - 0.15
24-hour urinary free cortisol (µg/24h)	**206.7**	8 - 70

The biochemical results of January 2015 (with pathological results reported in bold letters) were suggestive for ACTH-independent Cushing’s syndrome and excluded several potential differential diagnoses (e.g. primary hyperaldosteronism, and catecholamine excess).

The pathological biochemical results of April 2019 were instead suggestive for ACTH-dependent Cushing’s syndrome.

ACTH, adrenocorticotropic hormone; DHEA-S, dehydroepiandrosterone-sulfate.

In February 2015, the patient underwent successful laparoscopic left-sided adrenalectomy. Postoperatively, morning serum cortisol was < 5 µg/dl and glucocorticoid replacement therapy was initiated. The histopathological analysis revealed a well-circumscribed tumor, with foamy lipid-rich cytoplasm, a Ki-67 <1%, and no signs of malignancy (Weiss score: 1) (13). The analysis was compatible with a cortisol-producing adrenocortical adenoma. Slides from the histological investigations are exemplarily shown in **Figures 1B–D**.

Between February and May 2015, anti-hypertensive therapy could already be reduced to only one drug, and subsequent blood pressure levels were normal. Hydrocortisone was stopped in May 2015, taking into account a normalized adrenal function measured by an ACTH stimulation test and no clinical evidence for an ongoing glucocorticoid withdrawal syndrome.

**Figure 2:**
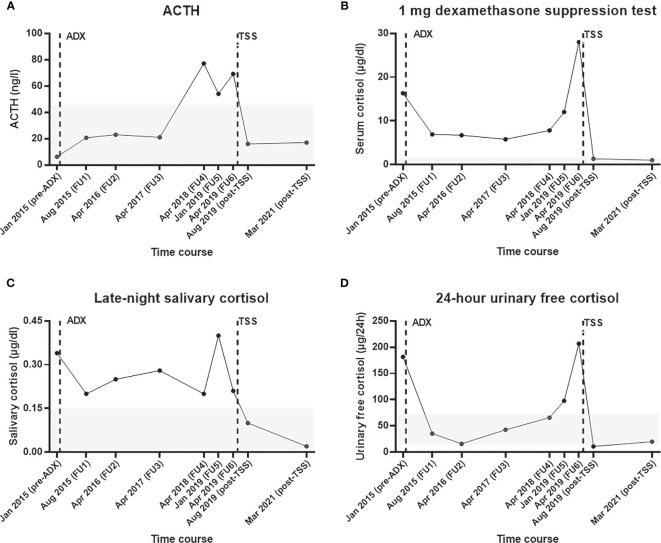
Selected endocrine parameters from initial diagnosis of adrenal Cushing’s syndrome until recovery after transsphenoidal surgery for Cushing’s disease. Course of **(A)** plasma ACTH, **(B)** serum cortisol during the 1 mg dexamethasone suppression test, **(C)** late-night salivary cortisol, and **(D)** 24-hour urinary free cortisol. The time of adrenalectomy (in February 2015) and transsphenoidal adenomectomy (in June 2019) are illustrated with vertical broken bars. The reference range of each test is shown as a grey area. ACTH, adrenocorticotropin hormone; ADX, adrenalectomy; FU, follow up; TSS, transsphenoidal surgery.

In August 2015, the patient reported a weight loss of 5 kg and an improvement of her general condition. The 1 mg dexamethasone suppression test (DST) and the late-night salivary cortisol (LNSC) results were remarkably lower than preoperatively, but not normalized (**Figure 2**). In contrast, 24-hour urinary free cortisol (UFC) was within the reference range and the ACTH was no longer suppressed. The patient was subsequently followed up for three years on an annual basis. The course of the four most relevant endocrine parameters (i.e., ACTH, 1 mg DST, LNSC, and 24-hour UFC) during our observation is provided in **Figure 2**. Two years after adrenalectomy, the antihypertensive therapy was discontinued and the patient did not take any medication at all. Additionally, the HbA1c was clearly improved (the lowest level was 5.5% in April 2017) compared to preoperative levels.

In April 2018, however, the patient reported symptoms suggestive for recurrent CS (i.e., proximal myopathy, moderate fatigue, and weight gain of 5 kg in 6 months) for the first time. Her general practitioner had already restarted anti-hypertensive medication because of uncontrolled blood pressure. Biochemical workup revealed recurrent glucocorticoid excess, surprisingly without suppressed ACTH (**Figure 2**). Due to the patient’s medical history, an abdominal contrast enhanced computer tomography (CT) scan was performed. This showed an inhomogeneous, hypodense, non-enhancing soft tissue mass of 2.3 x 1.6 cm in position of the removed left adrenal gland. For further investigations, we performed a [^123^I](R)-1-[1-(4-iodophenyl)ethyl]-1H-imidazole-5-carboxylic acid azetidinylamide imaging ((^123^I)-MAZA) whole body scintigraphy and single-photon emission computed tomography (SPECT)/CT. (^123^I)-MAZA is a tracer that binds specifically to the two adrenocortical enzymes 11ß-hydroxylase (CYP11B1) and aldosterone synthase (CYP11B2). However, these imaging methods did not show the supposed adrenal remnant at the level of the former left-sided cortisol-producing adrenal adenoma (**Supplementary Figure 1**). Furthermore, basal ACTH was above 50 ng/l. Further clinical and biochemical workup was delayed because the patient had the impression that her general condition was not severely affected.

In January 2019, she described that fatigue and myopathy were now more intense and that arterial hypertension was very difficult to control. At that time, the biochemical tests were once again clearly pathological (**Figure 2**). Accordingly, we suggested additional diagnostic and therapeutic measures, but she refused them and gave priority to a gynecological surgery.

**Figure 3:**
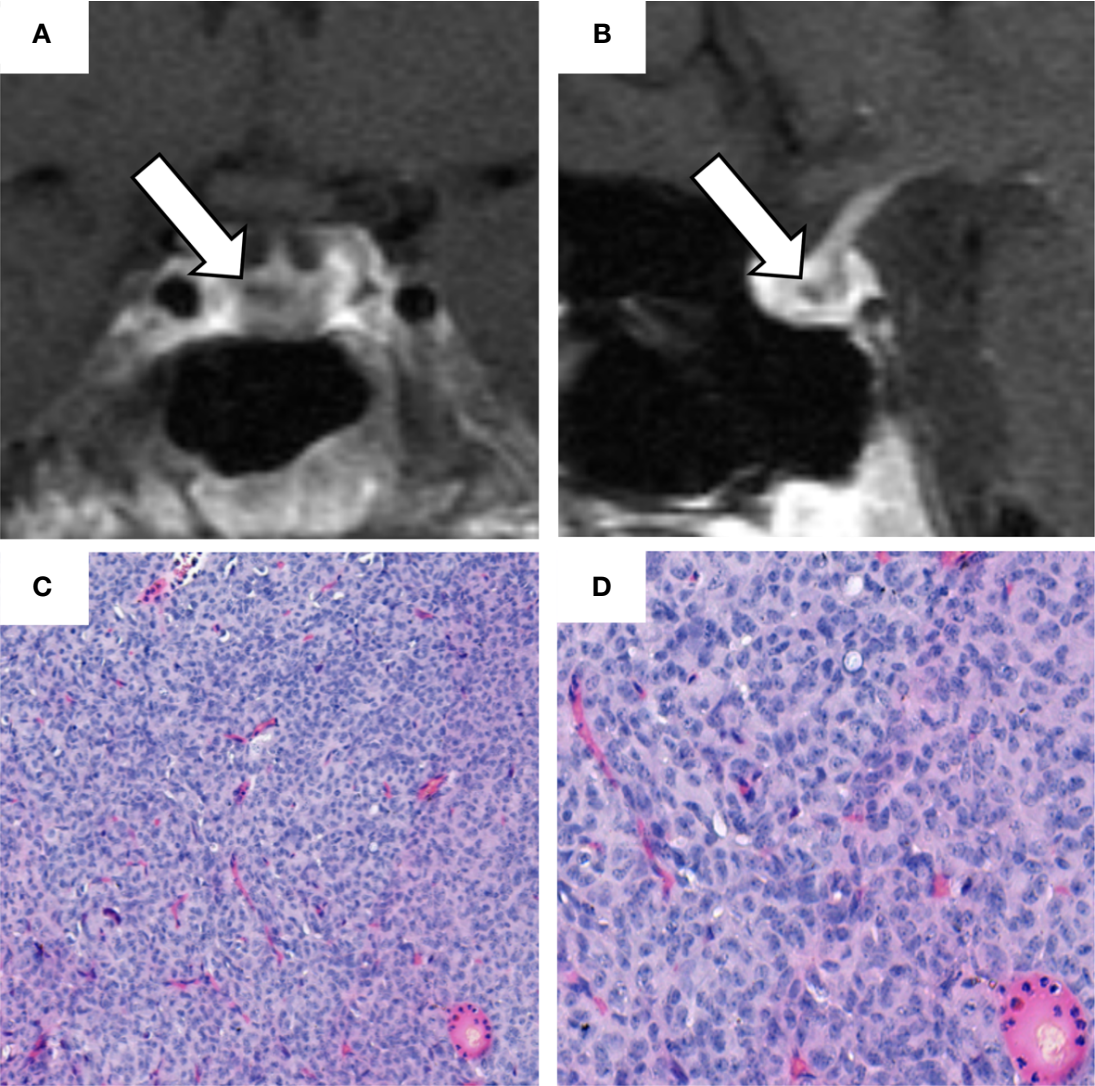
Sellar magnetic resonance imaging (MRI) performed in April 2019 and histological analysis of the pituitary adenoma. **(A)** coronal and **(B)** sagittal contrast-enhanced T1-weighted sellar magnetic resonance imaging. The white arrows indicate a hypointense focal lesion of 0.5 x 0.4 x 0.4 cm in the dorsal part of the adenohypophysis, reaching to the right side of the gland. This lesion was radiologically regarded as compatible with a microadenoma. **(C, D)** hematoxylin and eosin staining of the ACTH-secreting pituitary adenoma with: fragmented parts of a good differentiated epithelial tumor; cells present with medium size and oval nuclei (with an interspersed chromatin structure); occasional detection of nucleoli; no evidence of high mitotic activity; cytoplasm predominantly chromophobe. (H.E. 10x, 20x).

After successful removal of uterine leiomyomas in March 2019, we performed a comprehensive endocrine re-evaluation in April 2019. In a first step, ACTH-dependent CS was confirmed (**Table 1**). In a second step, dynamic diagnostic procedures were performed to establish the subtype of CS (i.e., pituitary vs. ectopic ACTH source). As recommended in such cases (1), both a corticotropin-releasing hormone (CRH) test and a desmopressin test were carried out, showing an ACTH-increase of more than 40% each (**Supplementary Table 1**). Furthermore, a pituitary MRI revealed a hypointense focal lesion of 0.5 x 0.4 x 0.4 cm in the dorsal part of the adenohypophysis compatible with a pituitary microadenoma (**Figures 3A, B**). In order to rule out the presence of a non-functioning pituitary adenoma and to confirm pituitary origin of the ACTH excess, a bilateral inferior petrosal sinus sampling was performed. This procedure revealed ACTH central to peripheral (IPS:P) ratios of 8.6 at baseline and 65.2 after CRH stimulation, indicating CD. According to these results, a central ACTH source was assumed.

The patient was, therefore, referred to pituitary surgery. In June 2019, she underwent successful transsphenoidal adenomectomy. The histopathological analysis described a 0.5 x 0.5 x 0.2 cm pituitary adenoma. The histological analysis (exemplarily shown in **Figures 3C, D**) and the immunohistochemistry revealed ACTH-expression in >50% of the cells, whereas staining for prolactin, thyroid-stimulating hormone (TSH), follicle-stimulating hormone (FSH), and luteinizing (LH) was negative. As an indirect hint for hypercortisolism, some Crooke cells were also identified in the normal adenohypophysis tissue (14).

Postoperatively, basal ACTH was <5 ng/l and cortisol levels were suppressed. Accordingly, glucocorticoid replacement was initiated. In the first postoperative follow-up 10 weeks after surgery (August 2019), an ACTH stimulation test revealed a persistent partial adrenal insufficiency (serum cortisol after 1 hour: 17.5 µg/dl), while the basal ACTH level was 16 ng/l. As reported in **Figure 2**, the biochemical workup (including 1 mg DST, UFC, and LNSC) indicated remission from CS.

Because we suspected the presence of a germline mutation and in order to discover potential driver mutations, whole exome sequencing analysis was performed (using tumor material from both the adrenal and the pituitary adenomas, with germline DNA from whole blood as a reference). The methodic of the analysis is described in the “Materials and Methods” section. Nineteen somatic mutations were identified in the adrenal adenoma, facing 16 somatic mutations in the pituitary adenoma (**Supplementary Table 2**). None of these mutations were shared, rendering independent development of both tumor entities. Among the observed somatic mutations, we identified a pathogenic somatic *CTNNB1* alteration (NM_001098209.2:exon3:c.133T>G:p.Ser45Ala; variant allele frequency (VAF) 33.6%) in the adrenal adenoma; in the pituitary adenoma, a pathogenic *USP8* mutation (NM_001128610.3:exon14:c.2152T>C:p.Ser718Pro; VAF 40.2%) as well as a pathogenic alteration in the glucocorticoid receptor gene *NR3C1* (NM_000176.3:exon5:c.1729_1735del:p.Trp577Argfs*15; VAF 31.7%) were observed.

The patient provided written informed consent to two disease-specific clinical registries and agreed in the current case presentation. The approval numbers from the local ethics committee of the University Hospital of Würzburg are 88/11 (for the European Network for the Study of Adrenal Tumors registry) and 85/12 (for the Network of Excellence for Neuroendocrine Tumors registry). In the last follow up (conducted in March 2021), the patient was in complete clinical and biochemical remission (**Figure 2**) and reported no signs and symptoms suggestive for Cushing’s syndrome.

## Materials and Methods

### DNA Extraction

DNA was isolated from snap-frozen adrenocortical adenoma sample with the Maxwell^®^ 16 Tissue DNA Purification Kit (#AS1030, Promega, Madison, WI, USA) and from formalin-fixed paraffin-embedded (FFPE) pituitary adenoma with the QIAamp DNA FFPE Tissue Kit (#56404, Qiagen, Hilden, Germany) according to the manufacturer’s instructions. Leukocyte DNA was extracted from whole blood using NucleoSpin^®^ Blood L (#740954.20, Macherey-Nagel, Düren, Germany), as previously described (15). The quality of DNA was analyzed by the Qubit^®^ dsDNA BR (Broad-Range) Assay Kits (#Q32850, Thermo Fisher Scientific, Waltham, MA USA). Tumor and leukocyte DNA was enriched with the Twist Human Core Exome Plus Kit (Twist Bioscience, San Francisco, CA, USA). Paired end sequencing with a length of 100 bps was performed on a NovaSeq 6000, 2 x 100 bp (CeGaT, Thüringen, Germany), according to the manufacturer’s protocol (yielding a coverage of 281x in the blood samples, of 525x in the adrenocortical adenoma, and of 284 x in the pituitary adenoma).

### Genetic Analysis

After an initial quality assessment using FastQC, v0.11.5 (Available online at: http://www.bioinformatics.babraham.ac.uk/projects/fastqc), adapters and low-quality reads were trimmed with TrimGalore, v0.4.0 (Available online at: http://www.bioinformatics.babraham.ac.uk/projects/trim_galore/) powered by Cutadapt, v1.8. The trimmed reads were mapped to the UCSC human genome (hg19) with BWA mem, v0.7.17 (16) before being sorted and indexed using Picard, v1.125 (available online at: http://broadinstitute.github.io/picard/) and SAMtools, v1.3 (17), respectively. Duplicates were marked with Picard. For coverage calculations and base quality score recalibration, GATK3, v3.5 and GATK4, v4.0.11.0 were used (18).

GATK3 and GATK4 were used for germline variant calling. Additionally, small germline indels were called using Scalpel v 0.5.312 (19). MuTect1, v1.1.410 (20) and MuTect2 (that is integrated in the GATK4 package),samtools (mpileup) with VarScan2, v2.4.111 (21) and Scalpel were used for somatic variant calling. All variants were annotated with ANNOVAR, v2019-10-24 (22) and considered if they were below a frequency of 2% in the databases 1000g2015aug_all, ExAC_nontcga_ALL, gnomAD_exome_ALL and gnomAD_genome_ALL (if the position is covered by at least 20 reads, and the alternative allele is covered by at least 8 reads and comprised at least 5% of the total reads). Each variant was visually verified using IGV, v2.7.2 (23).

## Discussion

To our knowledge, this is the first published case reported describing a histologically confirmed ACTH-dependent CS in a patient who had suffered from an ACTH-independent adrenal CS few years earlier. In our opinion, this case is remarkably not only from a clinical, but also from a pathophysiological perspective.

The major clinical challenge was to accept the unexpected, i.e. to agree to the diagnosis of CD after former adrenal CS. Of course, our first hypothesis was a relapse of adrenal CS, particularly as the abdominal CT scan report described an uncertain mass at the level of the formerly resected adrenal gland. At that time, the relatively high plasma ACTH was interpreted as part of the recovery process from adrenal CS. However, doubts arose when an adrenocortical remnant could not be confirmed by the (^123^I)-MAZA whole body scintigraphy and SPECT/CT. Therefore, the unclear mass was finally regarded as scar soft tissue. Recently, some authors reported assay-specific spurious ACTH levels leading to diagnostic and therapeutic obstacles (24). However, our analytical methodology did not change throughout the diagnostic process, making falsely elevated ACTH levels very unlikely.

It is certainly tempting to speculate on the starting point of ACTH oversecretion in our case. Postoperative improvement of both the patient´s clinical and biochemical condition seem to prove the development of CD many months after surgery for adrenal CS. As outlined in **Figure 3**, there was an increase in the ACTH and cortisol levels throughout the years despite initial normalization of UFC following adrenalectomy. The fact that 1 mg DST and LNSC only improved (but not normalized) may be explained by a physiologic/non-neoplastic hypercortisolism related to the obesity of the patient (25), but does not exclude an initial coexistence of a more pronounced adrenal CS and a - at that time - less relevant CD.

In the literature, the coexistence of the two subtypes of endogenous CS has already been described in few patients. Hernandez-Ramirez et al. (10) reported a case of ACTH-dependent and ACTH-independent CS coexisting in a patient with Carney complex. In this case, PPNAD developed 5 years after CD. In our patient, Carney complex was clinically unlikely due to the lack of typical signs and symptoms (such as pigmented lesions of the skin and mucosa, cardiac, cutaneous and other myxomas). Moreover, exome sequencing excluded a causative germline mutation of *PRKAR1A* but also of *MEN1*. With respect to the latter, Dal Verme et al. (11) reported the case of a patient who suffered from ectopic Cushing’s syndrome due to a thymic carcinoid 32 years after being treated for Cushing’s disease, possibly pointing to a *MEN1* germline mutation.

Schteingart et al. (26) presented a patient with suspected coexistence of CD and a cortisol-producing adrenal adenoma. However, histopathological analysis finally revealed an adrenocortical hyperplasia (as origin of hypercortisolism) along with a solitary non-functioning adrenocortical adenoma. Recently, Di Dalmazi et al. (7) described two cases with transition from ACTH-dependent to ACTH-independent hypercortisolism associated to micronodular adrenal hyperplasia and a catalytic α subunit of protein kinase A (*PRKACA*) somatic-mutation.

Two other case reports described persistent hypercortisolemia after transspehoidal removal of an ACTH-secreting pituitary microadenoma (8, 9). However, at least in one of these cases, the causative adrenal macronodule was already present at the time of the initial diagnosis of Cushing’s syndrome (8); in the other case, the adrenal mass was detected 10 months after pituitary surgery (making an ACTH-driven nodular autonomy instead of the consecutive occurrence of two independent Cushing subtypes very likely) (9).

Our own case, however, differs significantly from the already published cases. Firstly, in the two patients reported by Di Dalmazi et al. (7) and in the patient reported by Timmers et al. (9), CD was present before the adrenal CS and the initial stimulation of ACTH/Pro-opiomelanocortin (POMC) might therefore be pathophysiologically involved in the development of the adrenal hyperplasia. Secondly, in our case the histopathological analysis of the adrenal tumor clearly described an adrenocortical adenoma and no micronodular or macronodular hyperplasia. Thirdly, we performed whole exome sequencing of both adenomas and could clearly demonstrate that they are genetically distinct, whereas none of the previous reports could (dis)prove a similar situation.

*CNTNNB1* is a gene encoding for the β-catenin protein, which has a principal role in the Wnt signaling pathway. The role of β-catenin in the embryonic development of adrenal cortical cells and in the renewal of the adult adrenal cortex is well known (27). Additionally, the mutation of this protein and the constitutive Wnt pathway activation is reported in the context of the development of benign and malignant adrenal tumors (28–32). Although studies suggested that mutations in *CTNNB1* are more common in inactive adrenal adenomas (28, 29, 33), they have also often been reported in cortisol-producing adrenal adenomas (28, 29, 33, 34). The genetic analysis of the present adrenal tumor revealed a somatic mutation p.Ser45Ala of the *CTNNB1*-gene. This mutation has already been reported in the context of autonomous cortisol secretion (33, 34); of note, our current case was not part of these studies. Differently from previous reports, our case was associated with signs and symptoms of CS, which clearly improved after adrenalectomy. Moreover, as previously reported (28, 33, 34), a *CTNNB1*-mutation is associated with larger tumors, explaining the size greater than 40 mm of the adrenal adenoma in our case. Nevertheless, malignancy was excluded by a Weiss score of 1 and a Ki 67 index of <1%.

The exome analysis on the ACTH-secreting pituitary adenoma revealed a p.Ser718Pro-*USP8* somatic mutation. *USP8* is a gene which encodes for a protein named Ubiquitin specific peptidase 8 (35–38). This deubiquitinase enzyme removes the ubiquitin residues that physiologically target proteins for recycling or destruction. According to current knowledge, somatic USP8 mutations are present in about 24-40% of all CD cases (35–38). The most common mutation site of *USP8* in the context of CD has been found at the level of the exon 14 (NM_001128610.3), between the amino acids 713 and 720, in the 14-3-3 binding motif (36–38). In our case, exome analysis revealed a somatic mutation exactly in this region of exon 14 (p.Ser718Pro2-*USP8*). This mutation impairs the binding of 14-3-3 proteins (which have a regulatory role on *USP8*), thus increasing the deubiquitination of the Epidermal Growth Factor Receptor (EGFR), favoring its overexpression (36–38).

It is worth mentioning that the genetic analysis of the pituitary adenoma also revealed a frameshift deletion of the nuclear receptor subfamily 3 group C member 1 (*NR3C1*) gene that encodes for the glucocorticoid receptor. Somatic mutations of this gene have already been reported in 4 out of 64 (6.2%) ACTH-secreting pituitary adenomas analyzed by exome sequencing (38). While the mutation that we found now (NM_000176.3:exon5:c.1729_1735del:p.W577Rfs*15) has not yet been reported previously, the somatic mutations reported were all either inactivating point mutations or deletions. The recurring mutation-induced inactivation of the glucocorticoid receptor suggests that this process may be partly responsible for the manifestation of CD. This assumption is supported by analyses of the frequent inactivating germline polymorphisms showing that such disturbances in hypothalamic-pituitary-adrenal signaling homeostasis have important functional effects (39–41). Possibly, the distinct difference in cortisol levels before and after adrenalectomy could have influenced the glucocorticoid receptor turnover in the pituitary, resulting in increased glucocorticoid resistance and cell proliferation, and finally leading to the formation of an autonomous adenoma.

In conclusion, no association between somatic mutations of *CTNNB1* (in cortisol-producing adrenal adenomas), *USP8* and *NR3C1* (in ACTH-secreting pituitary adenomas) has been reported so far. To our knowledge, this is the first published case reporting two independent ACTH-independent and ACTH-dependent subtypes of CS developing in a single patient. Accordingly, the extremely rare event of recurrent hypercortisolism due to different subtypes of CS must be taken into account if corresponding (but unexpected) findings are observed during follow-up.

## Ethics Statement

The studies involving human participants were reviewed and approved by the local ethics committee of the University Hospital of Würzburg Approval numbers: 88/11 and 85/12. The patients/participants provided their written informed consent to participate in this study. Written informed consent was obtained from the individual(s) for the publication of any potentially identifiable images or data included in this article.

## Author Contributions

Clinical data were obtained by MD, MK, MF, and TD. Imaging data were provided by WS and AS. Tumor material was provided by SSc, RC, and VW. Genetic analyses were performed by BA and SA, and results were interpreted by SSb. MD and TD wrote the first draft of the manuscript which was then revised by all co-authors. All authors contributed to the article and approved the submitted version.

## Funding

This study was funded by the German Research Foundation (Deutsche Forschungsgemeinschaft (DFG); project numbers: 314061271 – CRC/TRR 205.

## Conflict of Interest

The authors declare that the research was conducted in the absence of any commercial or financial relationships that could be construed as a potential conflict of interest.

## Publisher’s Note

All claims expressed in this article are solely those of the authors and do not necessarily represent those of their affiliated organizations, or those of the publisher, the editors and the reviewers. Any product that may be evaluated in this article, or claim that may be made by its manufacturer, is not guaranteed or endorsed by the publisher.

## Supplementary Material

Supplementary Figure 1 (123I)-MAZA whole body scintigraphy and single-photon emission computed tomography performed in July 2018. **(A)** Whole body scintigraphy in LDR projection showing a physiological tracer uptake of the right adrenal gland (black arrow), **(B)** transversal, and **(C)** coronal (123I)-MAZA SPECT/CT fusion of the upper abdomen showing no tracer uptake of the inhomogeneous, hypodense soft tissue mass detected in the position of the formerly resected left adrenal gland (white arrows), but expected physiological tracer uptake of the right adrenal gland. ^123^I-MAZA, [^123^I](R)-1-[1-(4-iodophenyl)ethyl]-1H-imidazole-5-carboxylic acid azetidinylamide; CT, computed tomography; LDR, left side, dorsal view, right side; SPECT, single-photon emission computed tomography.

Supplementary Table 1 Dynamic tests for the differentiation of ACTH dependent Cushing´s syndrome performed at our endocrine ward in April 2019. Both the CRH and the desmopressin test indicated a pituitary ACTH source, whereas the 8 mg dexamethasone favored an ectopic ACTH source. Pathological parameters are presented in bold letters. ACTH, adrenocorticotropic hormone; CRH, corticotropin-releasing hormone.

Supplementary Table 2 Table of somatic mutations identified in the adrenal and in the pituitary adenoma. Detailed description of the somatic mutations identified in both adrenal and pituitary adenoma.
