# Supplementary figures and images for: Case Report: Consecutive Adrenal Cushing’s Syndrome and Cushing’s Disease in a Patient With Somatic CTNNB1, USP8, and NR3C1 Mutations
Source: Front Endocrinol (Lausanne). 2021 Aug 20;12:731579. doi: 10.3389/fendo.2021.731579 (PMC8417750; doi:10.3389/fendo.2021.731579)

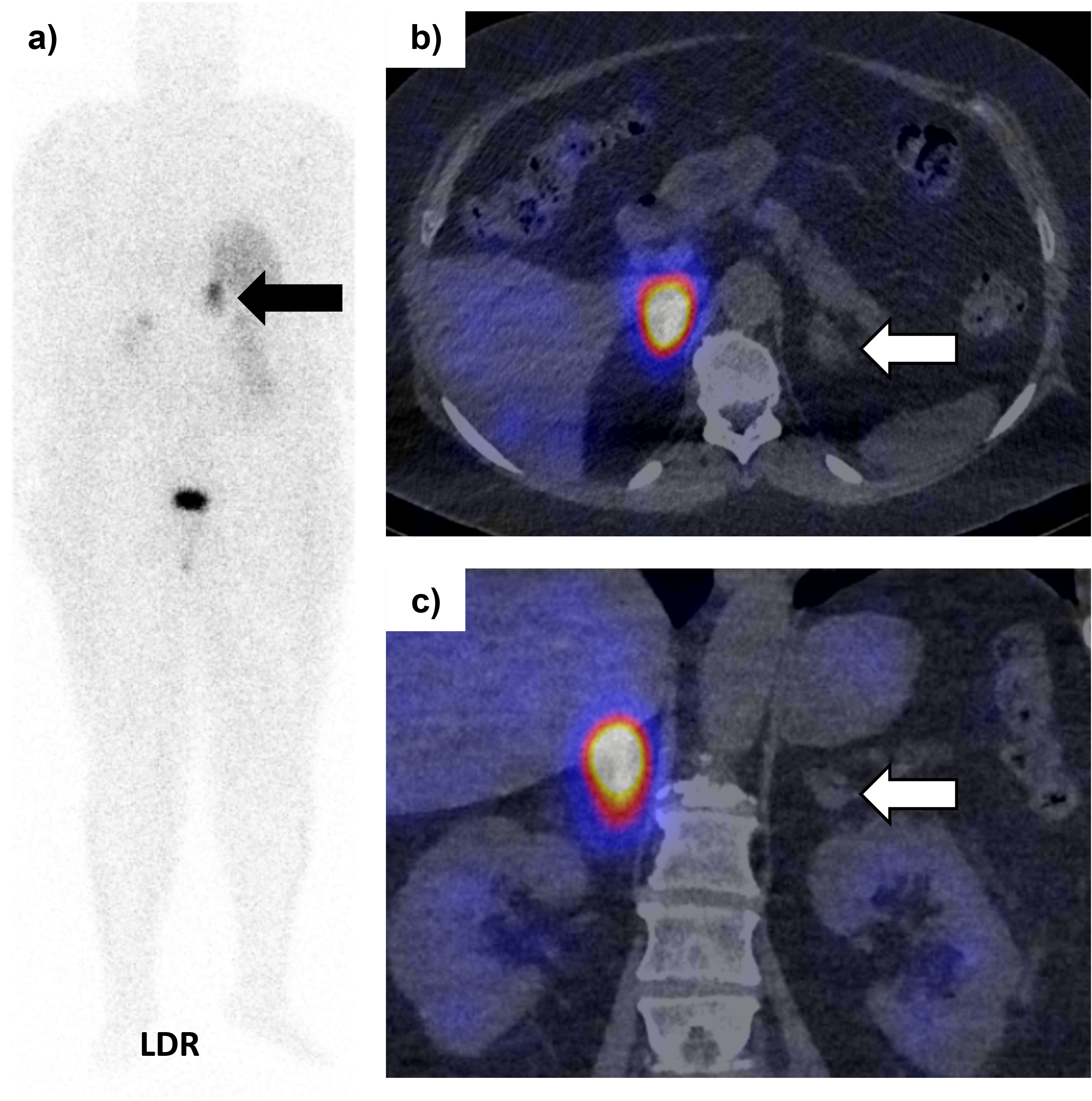

Supplement: Supplementary Figure 1 — (123I)-MAZA whole body scintigraphy and single-photon emission computed tomography performed in July 2018. (A) Whole body scintigraphy in LDR projection showing a physiological tracer uptake of the right adrenal gland (black arrow), (B) transversal, and (C) coronal (123I)-MAZA SPECT/CT fusion of the upper abdomen showing no tracer uptake of the inhomogeneous, hypodense soft tissue mass detected in the position of the formerly resected left adrenal gland (white arrows), but expected physiological tracer uptake of the right adrenal gland. 123I-MAZA, [123I](R)-1-[1-(4-iodophenyl)ethyl]-1H-imidazole-5-carboxylic acid azetidinylamide; CT, computed tomography; LDR, left side, dorsal view, right side; SPECT, single-photon emission computed tomography. [file Image_1.tif]
