# Supplementary material for: Case Report: Consecutive Adrenal Cushing’s Syndrome and Cushing’s Disease in a Patient With Somatic CTNNB1, USP8, and NR3C1 Mutations
Source: Front Endocrinol (Lausanne). 2021 Aug 20;12:731579. doi: 10.3389/fendo.2021.731579 (PMC8417750; doi:10.3389/fendo.2021.731579)
Supplement: Supplementary Table 1 — Dynamic tests for the differentiation of ACTH dependent Cushing´s syndrome performed at our endocrine ward in April 2019. Both the CRH and the desmopressin test indicated a pituitary ACTH source, whereas the 8 mg dexamethasone favored an ectopic ACTH source. Pathological parameters are presented in bold letters. ACTH, adrenocorticotropic hormone; CRH, corticotropin-releasing hormone. [file Table_1.docx]

**Supplementary Table 1. Dynamic tests for the differentiation of ACTH dependent Cushing´s syndrome performed at our endocrine ward in April 2019.**

| **High dose dexamethasone suppression test** | | | **Result** | | **Suppression from baseline (%)** | |
| --- | --- | --- | --- | --- | --- | --- |
| Serum cortisol after 8 mg dexamethasone (µg/dl) | | | 16.7 | | 29% | |
|  | | | | | | |
| **CRH test** | | | | | | |
|  | **0 min.** | **15 min.** | | **30 min.** | | **60 min.** |
| ACTH (ng/l)  [% increase from baseline] | 18.2 | 126.0  [592%] | | 156.0  [757%] | | 113.2  [522%] |
| Serum cortisol (µg/dl)  [% increase from baseline] | 13.9 | 20.2  [45%] | | 26.6  [91%] | | 30.7  [121%] |
|  | | | | | | |
| **Desmopressin test** | | | | | | |
|  | **0 min.** | **15 min.** | | **30 min.** | | **60 min.** |
| ACTH (ng/l)  [% increase from baseline] | 24.3 | 324.0  [1233%] | | 212.0  [772%] | | 100.0  [311%] |
| Serum cortisol (µg/dl)  [% increase from baseline] | 16.2 | 27.2  [67%] | | 33.2  [105%] | | 36.0  [122%] |
